# Cases of Indian Cholera

**Published:** 1831-10

**Authors:** Nathaniel Grant

**Affiliations:** Surgeon, formerly in the East-India Medical Service.


THE LONDON
### Medical and Physical Journal.

392, VOL. LXVII.] OCTOBER 1831. [64, *New Series.*

"For many fortunate discoveries in medicine, and for the detection of numerous errors, the world is indebted to the rapid circulation of Monthly Journals; and there never existed any work to which the Faculty, in Europe and America, were under deeper obligations than to the 'Medical and Physical Journal of London,' now forming a long but invaluable series."—RUSH.

### ORIGINAL PAPERS, AND CASES,
### OBTAINED FROM PUBLIC INSTITUTIONS AND OTHER AUTHENTIC SOURCES.

### INDIAN CHOLERA.

*Cases of Indian Cholera.* By NATHANIEL GRANT, Esq., Surgeon, formerly in the East-India Medical Service.

*To the Editor of the London Medical and Physical Journal.*

DEAR SIR: As at this time it may not be uninteresting, I beg to offer to you, for publication in your valuable Journal, a very short sketch of cholera as it appeared at two different times, where I had the treatment of the cases; and I subjoin a few of the cases, the notes of which were taken at the bed-side of the patients, and which give a very good idea of the fearful nature of the disease.

I am, dear sir, your most obedient servant,

NATH. GRANT.

21, *Thayer street, Manchester square;*
*September* 3*d*, 1831.

In September 1820, the H. C. ship Princess Amelia was lying at anchor in Whampau river, in China, in the middle of a large fleet of Company's ships, her crew in good health, when, without any warning, a young strong man was seized with symptoms of cholera, and was carried off in a few hours: the next day, two men died; the following day, two more. These were all young and previously healthy men, and their sudden deaths, and the peculiar and fearful symptoms accompanying, and even continuing after their dissolution, (viz. in some the muscles of the face, arms, and chest continued in action many hours after death,) produced among the rest of the crew a feeling of great dread: it continued carrying off two or three victims daily for eight days, when its violence as suddenly ceased, and all the cases afterwards were mild and tractable. In its mode of attack there was a good deal of variety: in some cases it began with violent pain about the umbilicus, weight on the præcordia, sometimes vomiting, often not; shortly after, cramps in the calves of the legs, fingers, or toes; sometimes a stiffness only of the muscles of the thighs or arms; after a time, the cramps ascended to the muscles of the abdomen, when the fæces and urine were ejected involuntarily; a sense of intense heat, and a fluttering pain at the pit of the stomach, shewed themselves; the muscles of respiration were generally last affected. In one or two muscular men, the progress of the cramp could be distinctly traced by the hard knots formed, as in the case of Mr. Delvin. In some cases, faintness and excessive debility were the first symptoms, as in some of the cases on September 18th, when strong healthy men, in a moment, felt their strength inadequate to support them, and fell down, their countenances immediately suffering a frightful change: in some men, with whose appearance I was perfectly familiar, the change was so great that I could not recognize them.

The pulse varied much in the different cases: in some, it was gone at the wrist a few minutes after the attack; in others, it was only feeble and very quick, sometimes hard; at times, when there were violent cramps, soon terminating fatally, it was scarcely altered from a natural and healthy state. When bleeding was used, it sometimes sunk after a little blood had flowed, and never rallied; in others, it rose and became soft, such cases proving favorable.

The violent cramps were always relieved by hard rubbing. In no case were the muscles of the face and neck affected.

In every case that could be traced, a thin watery purging was a precursory symptom. The matters vomited never had any intermixture with bile. Flatulence sometimes was very distressing. The senses were clear, generally, to the last, unless when dozed by the opiates.

In all the fatal cases, a very peculiar appearance of the fingers and hands shewed itself: the skin appeared shrivelled and bleached, as if it had been long soaking in water. There was no case in which this shewed itself that did not prove fatal.

Much pains were taken to endeavour to discover a cause for this visitation of the cholera, but nothing satisfactory could be elucidated. There was only one other ship, among nearly thirty, that suffered as we did, and she was moored at some little distance from us. We discovered that in the town of Whampan, and in some of the neighbouring villages, a disease, from description having some resemblance to cholera, was making dreadful ravages; and at the same time, on the borders of the province of Quang-Tong, the people employed to transport the tea across a chain of mountains separating the two provinces, were seized in great numbers, and their deaths were sudden. The season had been more than usually dry, and excessively hot.

### CASES.

Case I. Alfred Dutton, æt. twenty-five; Sept. 16th, 1820.

Half-past twelve, noon. Suddenly fell down from excessive weakness; face very pale and sunk; profuse cold sweats on face and breast; tongue a little foul; pulse eighty. Vomited a quantity of undigested food. An emetic was given to clear the stomach, and brought away a great deal of brownish stuff. This man had been, the previous evening, assisting in burying a messmate, and felt chilled; but he slept well, and felt himself quite well previous to the attack. Since yesterday frequent stools, and, by description, of brown colour.

Half-past one p.m. Cramps in the calf of his right leg; perspiring very copiously, and is quite cold; one stool just now, feculent, with some knots in it; dyspnœa; thirst.—℞. P. Opii, gr. ij. in pil. h. s.; and he drank a tumbler of warm congee (rice water), with Liq. Ammoniæ, ʒj.

Half-past two. Cramps increase: had him hard rubbed with hot rum, and then put into a hot bath, temperature 110°, in which he remained twenty-one minutes. Cramps left him; he is very drowsy; looks dejected, pale, and anxious. He was well wrapped up in blankets, and the opium pills repeated.

Half-past three. Feels easy; is getting warm; breathes easier; retains what he drinks; the fingers of left hand are slightly cramped.—Rep. Pil. Opii. Two or three stools have been passed involuntarily.

Five p.m. Seems better, and is warm.

Six p.m. The breathing difficult; pulse 140. Sumat pilul. Cal. gr. x., P. Opii, gr. i.

Seven p.m. Breathing very laborious, with violent heaving of the chest; the action of the heart irregular; pulse at wrist hardly perceptible; every part of his body warm and perspiring, over the stomach intensely hot; is quite insensible. A vein was opened, and eight ounces of blood flowed, coagulating firmly as it reached the cup: no effect from it. Is unable to swallow. Frictions all over with oil and laudanum.

He continued gradually to get worse till half-past seven p.m., when he died.

The body remained for some time warm and covered with perspiration.

Appearances on opening the body, ten hours after death: Muscles of abdomen fleshy and rigid; the intestines much distended with air; the omentum extended over all their surface, and embracing them closely; some of its vessels distended with blood. On removing the omentum, the intestines looked pale; the stomach pale, much distended with air, and fluid that had been drank; the internal surface natural. Throughout the course of the intestines, much distention, as in the stomach, but perfectly natural in their structure and appearance. Colon towards the rectum, and the rectum itself, contracted; coats not thickened, but no unusual appearances on being cut into. Bladder collapsed. Liver, no adhesions; rather large, otherwise natural. Gallbladder full of bile. Lungs natural; heart natural, very little blood in it.

II. William Kelly, æt. thirty; strong muscular man.

September 18th, half-past eleven A.M. Is just brought down from the maintop, where he had been at work on the rigging. Extreme faintness; great loss of strength; is sick, and has vomited; face and breast covered with cold clammy perspiration; pulse at wrist nearly gone; bowels have been deranged since last night, but felt perfectly well before the attack.—℞. Cal. gr. xx.; Ext. Opii, gr. ij. fiat bolus statim sd., and washed down with Inf. Sennæ and S. Sodæ. This was rejected, and cramps attacked the calves of the legs. V.S. ad ℥xviij. Blood flowed freely: immediately after he had a stool of brownish appearance, and singularly frothy. He was put into the warm bath, and the vein again opened, and bled to ℥xxxvi., without any effect. Cramps and vomiting continue; refuses medicine; and at four P.M. he died, four and a half hours from the first symptoms of the attack.

III. Francis Lee, æt. nineteen; stout healthy lad.

September 18th, twenty minutes to twelve, noon. Just brought down from deck: complains of faintness and extreme weakness; headach; face very pale and sunk, and with his breast covered with cold clammy sweat; pulse eighty, very feeble. Belly has been irregular for some days, but he was in perfect health, and on duty, at the moment of the attack.—℞. Infusi Sennæ, ℥ij. stat. It was retained, and he lay quiet till half-past one, when cramps came on in both arms and legs. Sumat instanter T. Opii, ʒij., and hot fomentations were applied till the bath was ready.

Two P.M. Put him into the hot bath for half an hour: spasms were relieved; when removed to his hammock, they returned.—Rep. T. Opii, ʒij. Stools passing involuntarily.

Half-past three. Laborious breathing; and a little after four P.M. he died.

IV. John Shilling, æt, twenty-six; strong man.

September 18th, half-past eleven. Suddenly great prostration of strength and faintness; vomiting; pain across the stomach and in the left breast; thighs feel very stiff; bowels have been irregular. ℞. Infus. Senna et S. Sodæ, which soon procured some yellow feculent stools, with curious stringy fibrous appearance; slight cramps in fingers. V.S. ad ℥xlv.; et sumat T. Opii, ʒij. very soon after, stools coming away involuntarily.

Three P.M. Is warm and perspiring; occasional cramps in his calves; face improves, and pulse, which was nearly gone at the wrist, rises. In a short time cramps came on with great violence: had him rubbed with hot rum, and put into the hot bath, with momentary relief. Being well wrapped in warm blankets, he was removed to his hammock, when the cramps came on with great violence, causing him to cry out loudly. His lower jaw sunk; breathing became laborious; and the contents of the bowels ran out, if turned in any direction. A wineglassful of brandy, with T. Opii ♏xc., was poured down his throat, and his body hard rubbed with hot rum. In about an hour he shewed symptoms of reviving. The stimulus, in small quantities, was frequently given, and the rubbing continued. He again got into warm perspiration, and his appearance improved. He continued so during the night, and this morning, (19th, six A.M.) feels and looks cheerful. Stools during the night passed involuntarily. ℞. Cal. gr. x., Ext. Opii, gr. i.

20th. Continues to improve. He had several more doses of calomel, and an occasional cathartic; and on the 25th was convalescent.

V. John Deverill, æt. twenty-four.

September 19th, two P.M. Suddenly felt himself unable to stand from weakness, and almost immediately was cramped in his thighs; has vomited. He complains of pain in the bowels, but the cramps in his thighs distress him most. Tongue very loaded; pulse very weak and quick. A wineglassful of brandy, with T. Opii ♏c.

Four P.M. Cramps relax a little, and again return with great violence. He retained the stimulant. Rep. stimulus.

Eight P.M. Has had sleep, and is in a warm copious perspiration; pulse rising. ℞. Cal. gr. x., Opii, gr. v.

20th, eight A.M. Tolerable night; occasional cramps; pains in lower belly; tongue loaded; no motion; thirsty; pulse sixty, soft. ℞. P. Jalapii, ℈i.; Calomel, gr. vi. fiat bolus quamprimum sum.

He speedily became convalescent.

There were many cases similar to this, where similar treatment, with occasional venesection, soon recovered them.

VI. William Stevens, æt. twenty-two.

September 19th, six A.M. Complains of excessive weakness; is giddy and faint; very pale and sunk countenance; cold sweats on breast and face; great pain in left side and across the stomach; pulse not to be felt at the wrist. He swallowed a wineglassful of brandy, with T. Opii ♏ lxxx. As no change was produced, it was repeated in twenty minutes. Half an hour after this, he complained of terrible sense of weight across the stomach, and difficulty of breathing; pulse can hardly be felt, and is irregular.

V.S. ad ℥xlv., and gave him some hot punch, and had him wrapped in warm blankets.

Half-past ten. In copious warm perspiration, but he says he feels cold; slightly cramped. Mulled port wine, with volatile alkali in it for drink.

Four P.M. Feels easy; is warm and perspiring.

Seven P.M. Is quite easy; countenance cheerful. ℞. Infusi Sennæ et S. Sodæ.

20th, seven A.M. A tolerable night; no cramp; pulse fuller; one motion, copious, liquid, and dark coloured. Sumat bolum catharticum, and next day was convalescent.

VII. Seth Thompson, æt. fifty.

September 21st. Since yesterday morning has had a frequent watery purging, attended with griping; great depression of strength: at present feels cramped in his toes and fingers; countenance much sunk; pulse nearly gone at the wrist; tongue loaded and tremulous. A tablespoonful of brandy, with T. Opii ♏xx., to be taken every half hour. Sumat pilulam, Cal. gr. iij., secundâ quâque horâ.

22d. Purging diminished; still cramped in different parts of the body. The stimulant and the calomel pills are retained. He lies easy, but is very low, and appears sinking. To continue the stimulant and pills.

23d. Sinking fast. Seven P.M., died.

VIII. Samuel Cocherill, æt. eighteen.

September 22d. Was called to see this man at two A.M.; found him lying on the damp deck, quite cold; feels cramped in the legs, and a severe drawing-in pain in his stomach; face and breast covered with cold clammy perspiration; his countenance dreadfully altered; eyes sunk and hollow; face of a livid hue; pulse at the wrist gone. He was put into a warm bed, and employed people to rub him hard with hot rum: he swallowed immediately a bolus of Calomel gr. x., Opii gr. i., and a wineglassful of brandy with T. Opii ♏lxxx.: he retained these a short time only. He continued occasionally taking the stimulant, and warmth was kept up around him; but he never rallied, and at nine A.M. he died.

IX. Mr. Delvin, æt. thirty-two.

September 24th, five A.M. Called to see him, and found him with violent cramp in the calf of his left leg: he said that during the night purging had come on; he had been out frequently, and it was in an attempt to get into his cot that the cramp seized the leg. Pulse at this time was good. Sumat statim, a wineglassful of brandy, with T. Opii m. c. Soon after, cramp seized the calf of the other leg, alternated from one to the other, and then gradually ascended to the muscles of the thigh: the swelling and cramps could be distinctly traced along some of the muscles of the thigh; at length they seized the abdominal muscles; the intestines seemed at the same time to be affected: he screamed out with the pain.

After a time he cried out that he felt his heart cramped, and in a few minutes he became insensible: pulse at this time gone at the wrist and temples, breathing laborious; and at ten A.M. he died.

The only thing that gave any relief during his dreadful sufferings was hard rubbing.

X. John Tyler, æt. thirty.

September 27th, twelve, noon. Called to see him: complains of violent pain across the epigastrium, faintness, and great debility; countenance much sunk and very pale; cold sweats on face and breast; pulse fluttering, and nearly gone at the wrist. Sumat instanter bolum ex Cal. ℈i., Opii gr. ij. He took a wineglassful of brandy, with T. Opii ♏xl., and retained both.

About one P.M. cramps came on in his legs and arms. No appearance of reaction: he continues cold, although well covered up, and he says he feels warm; vomited once or twice; very thirsty. To be rubbed where cramped. He has taken another wineglassful of the stimulus. Fæces are passing involuntarily. Soon after he fell into a dose, and reaction seemed about to take place; but in a short time cramps came on with redoubled violence, and giving great pain. The stimulus, warm drinks, and hard rubbing were continued, and warm fomentations to the stomach, but in vain: at nine A.M. he died.

XI. John Downie, æt. forty.

September 28th, two P.M. Called to see him: he complains of severe pain in his stomach; legs and thighs cramped; countenance not much changed; pulse good and full; tongue foul; no vomiting; spirits much dejected. V.S. ad ℥xxiv. He took a wineglassful of brandy, with T. Opii m. lx., and a tumbler of hot conjee water, and was put into his hammock well covered up with blankets. The cramps continuing, in a quarter of an hour the stimulant was repeated. No vomiting, and very little thirst. Two hours after this he swallowed Calomel ℈i.

Nine P.M. After lying quiet for nearly two hours, he suddenly started up, and vomited a full quart of pure blood, which coagulated on the deck: he said he felt no warning of this whatever, nor did it make any alteration in his feelings afterwards; pulse very weak.

29th. Slept indifferently; complains of pain in his belly; no motion.—℞. Calomel gr. x.

Eight P.M. Easy all day. Vomited once during the day, after drinking some tea; one or two scanty darkcoloured stools; ischuria. Foment the lower belly; rep. Cal. gr. x.

A few cathartics afterwards completed the removal of his symptoms, and on October 4th was convalescent.

In July 1822, the H. C. ship Sir David Scott was lying at the new anchorage off Saugor Island, Bengal, where she had already been two months, and was preparing to proceed on her voyage to China, her crew in perfect health, when, on July 28th, a poor old man was seized with cholera. He had been ill for some hours before I was sent for, and, when I saw him, life was ebbing fast: there was no pulse, the body cold, and he was scarcely able to tell his complaints. Stimuli, external and internal, were used without effect: he died in ten hours from the attack.

No more cases occurred that day; but next evening a fine young man was seized: he also died in ten hours. The next day another case occurred, which died in twelve hours. Next day, ten men were seized, of whom two died, eight recovered. The next day, thirteen fresh cases, of whom five (all young, strong, and previously healthy men,) became the victims, after a few hours of suffering. The next day, eight fresh cases, of whom one died. Next day, four, two of whom died. After this a great many cases occurred, but much less severe, and none of them proved fatal.

The modes of attack did not vary much: almost invariably the first symptom that drew the attention of the patient was a watery purging; this they seldom complained of till vomiting, pains in the stomach, or cramps, frightened them; and at this time, in most cases, the countenance had undergone a considerable change; there was a peculiar sunk appearance around the eyes; great pallor, and in many, tremors of the lips; almost invariably the tongue was, at the very outset, much loaded with a thick white, sometimes brownish, crust. The skin in general was quite cool, often cold, and covered with clammy sweat, particularly on the breast; the pulse, in very many cases, when the patient first made known his complaints, was not to be felt at the wrist; in other cases, the pulse was nearly natural.

The cases of Henry Talbot and William Taylor shew the dreadful rapidity with which this fearful disease sometimes cuts off its victims; and also the uselessness of any attempt to check its progress.

In some cases, the first complaint was of pain in the left breast, and after a time the usual symptoms followed: these cases generally recovered. A sense of burning heat in the epigastric region, at the time that the body was quite cold, was a common symptom. On applying the hand to the pit of the stomach in some cases, the feeling of heat was intense. Cramps formed part of the symptoms in every case, but were no criterion of the violence of the disease; for, in many of the fatal cases, they were not so severe as in cases comparatively milder, and which recovered: sometimes the fingers and toes were the only parts affected; at other times, the muscles of the abdomen, legs, or arms. I remarked here, as in China in 1820, that in no case were the muscles of the face, neck, or back affected. Vomiting was a very general symptom, although there were several cases where it did not occur; in some it amounted merely to retching, in others any thing swallowed was instantly thrown up. The senses were generally unimpaired.

With regard to the state of the urinary organs, on which much stress is now laid as characterizing this disease, I regret not having attended more to it. The violent and fatal cases, ran their course so rapidly that the absence of urine did not strike the attention. I find in several cases that ischuria is mentioned as a symptom, and that it was relieved by topical warmth. In the only dissection I had an opportunity of making, (for the cases accumulated so quickly, and required such unceasing watching, that I had no time for inspection of the bodies,) I find the bladder was in a state of collapse.

In searching for a cause of this visitation of cholera, there is nothing to be found in the state of the atmosphere, as indicated by the thermometer or barometer, that will assist us; for there was no kind of change from what had previously existed: the heat was not greater than usual; thermometer from 78° to 86° F. The ship had been in its place of anchorage for two months, and during that time the crew had enjoyed perfect health: the breezes at that time always blew from seaward, and were cool and pleasant; there was no cholera in Calcutta, nor any where in our neighbourhood: it came upon us like a thunderclap. That it proceeded from some cause which had a general influence over the whole of the ship's company, but some more powerfully than others, I have reason to believe, as there was a great and an immediate change in the looks of the people, which might, in some degree, be occasioned by the fears that pervaded their minds: but there was something more than that; for, on looking at the tongues of those otherwise in health, they were invariably much loaded, usually covered with a thick white crust.

The alteration in the action of the bowels, after the disease had shewn itself, was sometimes very remarkable: in Talbot's case for instance, at one A.M. he had a copious, dark-coloured feculent stool, and at four A.M. he was passing a whitish milk and water-like stuff.

Fear seemed to add much to the destructive power of the disease, and I remarked the same circumstance here as in China, in 1820, with regard to its cause: after its first accession, it seemed to gather strength and violence to a certain point, and then decreased: thus, for the first three days, one man daily was seized; on the fourth day, ten; on the fifth, thirteen; on the sixth, eight; on the seventh, four. For two days there were no seizures, and afterwards the cases, with one exception, were mild; the disease was certainly not contagious.

With regard to the method of cure, I found it necessary to vary it in different cases. In those where extreme prostration of strength was the first and most alarming symptom, when there was no pulse at the wrist, and deathly coldness all over, I found the only chance of relief consisted in employing every means to keep up the warmth of the body: warm bottles were applied to the feet, fomentations to the stomach and belly, hard frictions to the limbs, and frequent doses of a stimulating mixture, consisting of one or two tablespoonsful of brandy, with from thirty to one hundred drops of laudanum; and if the stomach could retain any thing, warm brandy toddy, or wine mulled with plenty of spice, and some volatile alkali. The patients always called for cold drink, and frequently would not taste the warm; but I always repented gratifying this propensity, for cold drinks increased their sufferings. If by the means above mentioned reaction took place, and the circulation came back freely to the surface, a vein was opened, and bleeding, sometimes to a large amount, practised: generally the pulse rose and softened as the blood flowed. They were then covered well up, and a warm potion given to promote diaphoresis. Usually they slept for some hours, and, on awaking, a large dose of calomel, conjoined with some opium and P. Antimonialis, was administered; and in the morning a laxative, which, bringing away copious dark-coloured stools, put a period to the disease.

Bleeding, although a most powerful means of relieving the sufferings in this dreadful disease, requires much prudence to direct its use: where it is to be useful, it requires being used to a great amount; in some of the cases which follow, the bleeding seemed to have the effect only of accelerating death. In the case of Edward Riley, fifty-two ounces were lost: it gave relief to the cramps, but the pulse never rose, and he died in ten hours. Thomas Gooldacre, young, stout, and plethoric, with pulse quick and oppressed, was bled to twenty-five ounces, grew faint, pulse sunk, and never rose again. In the case of Parker, the pulse was rising and beating hard, when a vein was opened; after losing fourteen ounces, such an appalling debility came on, and the pulse sunk so much, that I was glad to stop the flow of blood as quickly as possible: this man recovered.

The warm bath was certainly very efficacious in removing or relieving the spasms; but it was often very inconvenient to put the patient into it. Blankets wrung out of boiling water were an excellent substitute: hard frictions, either with warm flannel, the warm hand, or heated rum, were of great use.

Opium, either in the form of pill or conjoined with brandy and peppermint water, was much used. A tablespoonful of the following mixture almost invariably checked, for a time, the irritability of the stomach: ℞. Brandy, ℥iijss.; Tæ. Opii, ʒss.; Aq. M. pip. ℥iv. M.

Calomel was very generally used, more with the intention of meeting the future symptoms of the disease than allaying the existing, although, in large doses, it seemed to tranquillize the stomach and bowels, when nothing else would. Conjoined with opium and Pulv. Antimonialis, it acted often like a charm. I found there was so great a disposition to dysentery left by the cholera, that, in most cases, I considered it prudent to touch the mouth with calomel before dismissing my patient.

CASES.

Case I. Thomas Francis, æt. sixty.

July 28th, five A.M. Called to see him, and found him complaining of severe cramps in his belly, legs, and thighs, making efforts to vomit, and passing stools involuntarily: face much sunk, body cold, and breast covered with cold clammy sweat; pulse at the wrist very feeble; and he had just strength to say he had been in this state for some hours. He was immediately wrapped in warm blankets; warm fomentations to the belly, and hard frictions to the limbs. Sumat instanter haustum, ℞. Tæ. Opii, m. lxxx.; Ætheris Sulph. ʒi.; Spt. Ammon. Aromat. ʒi.; Aq. Menth. pip. ℥iss. M. Bibat cyathum vini calide et si perstat vomitus sumat cochleare unum misturæ sequentis semi quâque horâ. ℞. Brandy, ℥iv.; Tæ. Opii, ʒss.; Aq. M. pip. ℥iij. M.

The vomiting was checked by the stimulant, and the cramps kept down by friction, but warmth was never restored. He died at four P.M.

II. Edward Riley, æt. twenty-four.

July 29th, eight P.M. Constant vomiting; intense pain in the stomach; occasionally feels cramped in the belly; appearance sunk; pulse feeble; hands and feet cold; for some hours has been much purged. Bled to ℥xij., and swallowed a wineglassful of brandy, with Tæ. Opii m. xx., and warm fomentations to the abdomen.

Nine P.M. Cramps have left the bowels, and were now very violent in his limbs; pulse very feeble and oppressed; hands and feet have the shrunk appearance of having been soaked for a long time in hot water. Rep. venæsectio ad ℥xl.: he bled freely; cramps were removed. Continue fomentations and frictions, and every half hour a spoonful of the stimulating mixture prescribed for Francis; warm punch for drink.

For some hours he was free from cramps, but nothing like reaction took place. He slept a little: the cramps returned; stools passed involuntarily, and nearly white; one stool, in particular, resembling curds and whey; pulse never rose. The medicine was continued every half hour. At two A.M. next day, breathing became laborious, and at six A.M. he died.

III. Thomas Gooldacre, æt. twenty-one.

July 30th, six P.M. For some hours has been purged, with griping, and took a dose of salts: stools whitish; nausea; pain at the pit of the stomach; slight cramps in belly and limbs; face pale; pulse very quick: he is much frightened. Sumat instanter bolus ex Calomel. gr. x., P. Opii gr. ij.

Eight P.M. Cramps increase in severity; stools frequent and white; has vomited once or twice; appearance much sunk; breast and extremities cold, and covered with profuse cold sweat; pulse quick and oppressed. Venæsectio ad ℥xxv.; rep. bolum ex Cal. et Opii; warm applications to the stomach and extremities; warm brandy and water for drink; every half hour a tablespoonful of the stimulating mixture.

Ten P.M. Medicines retained; extremities are warmer; face more sunk, and fingers bleached; violent cramps at times, always relieved by frictions; pulse much weaker.

Twelve P.M. Delirious and very restless; pulse not to be felt. At six A.M. he died.

IV. Thomas Henney, æt. thirty.

July 31st, half-past eight A.M. For some hours has been purged and griped; stools whitish; pain in stomach. Bolus ex Calomel. gr. x.; P. Opii, gr. i.; P. Antim. gr. iv. M. Fomenta calida, et cochleare unum Mist. stimulantis.

Half-past eleven A.M. Some cramps in the calves of his legs; pulse quick, and rather full; vomiting. Venæsectio ad ℥xxiv. After the bleeding he felt warm, and the pulse did not sink.

Half-past twelve. Cramps very severe: constant vomiting; face much sunk; pulse hard and quick. Rep. venæsectio ad ℥xvi.; sumat pilulam ex Calomel. gr. x., P. Opii gr. ij.; continue the stimulating mixture.

Two P.M. Much worse; face sunk; pulse gone at the wrist; fingers bleached. Continue stimulus. Four P.M. he died.

V. William Rice, æt. twenty-two.

July 31st, two P.M. Has been purged all day: complains now of pains across the epigastrium; slight cramps; pulse quick and weak. Sumat pilulam ex Calomel. gr. x., P. Opii gr. ij.; et cochleare Mistura stimulantis, unâ quâque horâ. Fomenta calida.

Four P.M. Cramps continue; retching. Rep. pil.; cont. mist. During the night, violent vomiting came on; cramps became more severe: great thirst, and he drank considerably of hot rice-water, with brandy in it. Strength gradually sunk, and at seven A.M. next morning he died.

This man, as well as Thomas Henney, were dreadfully frightened.

VI. James Parker, æt. twenty-five.

August 1st, nine A.M. Has been purged during the night, with much griping. Sumat Sulphatis Sodæ ℥i. statim.

Eleven A.M. Cramped in thighs and legs: twice freely purged since he took the salts; pulse very much fallen. Sumat pil. ex Calom. gr. x., P. Opii gr. i.; Mistura stimulantis, ℥ss. Fomenta calida abdomini.

Three P.M. No relief: constant cramp; countenance sunk; pulse has risen a little, and beats hard. Venæsectio ad ℥xiv.; continue stimulus. The pulse sunk during the bleeding, and no relief from it.

August 2d. During the night the stimulating mixture, in table-spoonfuls, was occasionally given; the warm fomentations continued. He rallied a good deal, and is now much better. Two pills of calomel and opium taken; rep. pilula; arrowroot and wine pro re nata.

3d. No cramps; stomach is irritable; mouth very slightly affected. Sumat sextâ quâque horâ pilulam ex Cal. gr. iij.; P. Opii gr. $\frac{1}{3}$; Pulv. Antimon. gr. i.; nourishing diet.

4th. Improves: mouth tender.

5th. Tongue much loaded. Omit pil., et sumat Infusi Sennæ et S. Sodæ ℥iij.

He continued improving, and in a few days was convalescent.

VII. John Small, æt. thirty.

July 31st, five A.M. Feels himself cramped; neither purging nor vomiting. Sumat pil. ex Cal. gr. x., P. Opii gr. i.

Ten A.M. Sumat Sulphatis Sodæ, ℥i.

August 1st. Has been freely purged, and has no complaint.

2d, eleven P.M. During the day was very well; but towards evening severe gastric pain came on, with retching and watery purging; feels himself cramped in the calves of the leg; tongue loaded; pulse full and strong. Venæsectio ad xxx.; bibat ex aqua hordei calida diaphoresim fovere.

3d. Slept well after the venesection, and has no complaint today but debility. ℞. Inf. Sennæ et S. Sodæ ℥iij.

VIII. John Underwood, æt. thirty-two.

July 31st, eleven A.M. Suddenly felt his right thigh stiff; is sick, and has pain in the stomach. Cal. gr. v. cum Inf. Sennæ et S. Sodæ, ℥ij.

Two P.M. Feels cramped in the thigh. Sumat pil. ex Cal. gr. x., Opii gr. ij.

August 1st. No cramps since the exhibition of the pill. Cont. pilula quartâ quâque horâ.

2d. Mouth sore; no complaint.

IX. Thomas Argent, æt. twenty-five.

August 1st, two A.M. Cramped all over; vomiting; pain in the stomach; pulse sunk. Sumat pil. ex Cal. gr. x., Opii gr. i.; Misturæ stimulantis cochleare unum frequenter; fomenta calida abdomini.

Six A.M. Vomiting allayed; pulse risen a little. Cont. pil. secundâ quâque horâ donec ptyalisum provocat.

2d. Still occasionally cramped; bowels freely moved, stools whitish. Cont. pil. sine Opio sexta quâque horâ.

3d. Mouth slightly tender; stools of dark colour. Sumat quartâ quâque horâ pil. ex Cal. gr. iij., P. Antimonialis gr. ss., P. Opii gr. $\frac{1}{6}$.

5th. Free ptyalism; no complaint.

There were many such cases as the above; and it may be thought that the treatment was more active than symptoms required; but it should be recollected that the cholera was then at its height, and many patients dying daily with symptoms quite similar.

X. —— Bondo, æt. thirty-four.

August 1st, ten A.M. Says he has been ill some hours: his countenance much sunk; pulse scarcely to be felt; body and extremities cold; is vomiting, and has been severely purged; stools white; pain in stomach; cramped in the thighs and abdomen. Sumat bolum ex Cal. ℈i., P. Opii gr. ij., cum cyatho vinoso Mist. stimulantis; fomenta calida. Warm ricewater, with brandy, for drink. He continued vomiting and cramped, and at four P.M. died.

XI. William Stevens, æt. twenty-six.

August 1, two A.M. Has vomiting; purging of a watery, whitish matter; cramps in abdomen and legs; pulse much sunk; countenance greatly altered. Pil. ex Cal. gr. x., Opii gr. ij., cum cyatho vinoso Mist. stimulantis; fomenta calida per totum corpus; frictiones; warm brandy and water per potu. Rep. pil. et mistura unâ quâque horâ.

Six A.M. In every way worse: stools passed involuntarily. Pergat in usu medicamentum, &c. Eight A.M. he died.

This man was greatly under the influence of fear, and had been a hard liver.

XII. William Taylor, æt. forty.

August 1st, ten A.M. Suddenly, while on duty on deck, became pale and sick, with an inclination to vomit. Pil. ex Cal. gr. x., Opii gr. i., P. Antim. gr. i.; and took a glassful of brandy, with T. Opii m. xxx.

Eleven A.M. Severe cramps in the abdomen and thighs; pulse scarcely to be felt. Sumat Cal. ℈i., P. Opii gr. ij. Repeat the brandy and opium; the warm bath; and warm brandy and water for drink.

Two P.M. No improvement: fingers looked bleached. Cont. medicamenta. Five P.M. he died.

XIII. Henry Talbot, æt. twenty-three.

August 1st, one A.M. Suddenly awoke from sound sleep, with cramp in the right thigh: hard friction removed it immediately. When I came to him, it was gone; his pulse natural; countenance the same, and he said he was quite well. He became sick, and vomited a great quantity of undigested food, mixed with bilious-looking stuff; almost immediately after, he felt inclined to go to stool, and passed, with great force, a copious dark-coloured stool, mixed with some undigested food. He said he was quite relieved, and would again go to sleep. Sumat pil. ex Cal. gr. x. Opii gr. i., cum Mistura stimulant.

Four A.M. He fell into a comfortable sleep, and has just awoke with cramp in his thighs: his countenance is dreadfully altered; pulse much sunk; severe cramps; vomits; and has passed a white stool. Sumat Cal. ℈i., Opii gr. iij.; occasionally a small wine-glassful of brandy, with twenty drops of T. Opii.

Six A.M. No favorable change. Pil. ex Cal. gr. x., Opii gr. i. secundâ quâque horâ; cont. brandy and T. Opium; warm wine for drink.

During the day, he appeared at one time to rally a little: his extremities were warm, and pulse rose. He was changed into a clean warm bed, and the medicines continued: but his extremities again cooled, pulse sunk, cramps again came on, and at four P.M. he died.

This man was young and remarkably healthy, regular in his habits, and in perfect health when he went to bed. When I first saw him, as his appearance and pulse were natural, and he assured me he was very subject to cramp, I did not bleed; which I much regretted, for, when I saw him three hours after, his pulse was so sunk that I dared not bleed. In this case, as in many others, although the breast and extremities were rigidly cold, he complained of burning heat over the stomach, and it was to the feel perceptibly very hot.

XIV. John Thompson, æt. thirty-five.

August 2d, four A.M. Gastric pain, vomiting, and purging; body cold; pulse nearly gone at the wrist; countenance sunk; thighs cramped. Sumat Calomel ℈i. instanter; Mist. stimulantis cochleare unum frequenter. Fomenta calida et frictiones.

One P.M. Easier: vomiting abated; cramps easier; pulse a little risen; purging continues. Sumat pil. ex Cal. gr. x., P. Opii gr. i. quartâ quâque horâ.

3d. Feels much better: purging stopped; tongue loaded. Omitt. pilula. Sumat Inf. Sennæ et S. Sodæ ℥iij.

XV. Mr. Kain.

August 2d, mane. Complains of watery purging; severe gastric pain, and across the umbilicus; has vomited; tongue much loaded; pulse good. Pil. ex Cal. gr. x., Pulv. Antim. gr. iij.

Vespere. Still pain, purging, and vomiting; pulse ninety-six, full. Venæsectio ad ℥xxx.; rep. pil. ex Cal. et P. Antim. quarta quâque horâ.

3d. Greatly relieved by the bleeding; slept well after it; stomach retains the calomel; stools mucous and mixed with blood; pulse soft. Cont. Cal. et P. Antim.

4th. Mouth sore; stools dark coloured, more copious and free; no pain; tongue less loaded. Sumat Inf. Sen. et S. Sodæ ℥ij.

5th. Freely purged; no complaint but the mouth.

XVI. John Knox, æt. thirty-five. August 3d.

This man has been many months an invalid, at first from pulmonary complaints, then scorbutic, and had lately been wasting away daily, and was reduced much; but still appetite continued good, and bowels regular. Today a watery purging and vomiting came on, with cramps of the thighs: he was almost immediately in extremity, and in the evening died.

XVII. —— Estherbrooke, æt. twenty-three.

August 3d, A.M. During the night was seized with vomiting, purging, and cramped all over: his body quickly became cold, and covered with clammy perspiration; pulse nearly gone at the wrist; tongue much loaded. Sumat instanter haustum ex Æther. Sulph. ʒij., T. Opii ʒiss.; fomenta calida per totum corpus.

Some time after, has took a bolus of Cal. ℈i., Opii gr. ij., and frequently a tablespoonful of the stimulating mixture. No reaction has took place, and at twelve, noon, he died.

